# Application of AI in predicting postoperative infections using routine blood parameters

**DOI:** 10.6026/973206300214271

**Published:** 2025-12-15

**Authors:** Angshuman De, Vasantavada Venkata Satya Sai Preeti, Mukul Singh, Mukesh Kumar Patwa, Niyati Pandya, Amrit Podder, Parth Jani, Chandan Gogoi

**Affiliations:** 1 Department of Biochemistry, Ramakrishna Mission Seva Pratishthan (Sishumangal Hospital), Kolkata, West Bengal, India; 2 Department of Community Medicine, Fakir Mohan Medical College and Hospital, Balasore, Odisha, India; 3 Department of General Surgery, All India Institute of Medical Sciences, Gorakhpur, Uttar Pradesh, India; 4 Department of Microbiology, ASMC Gonda, Uttar Pradesh, India; 5 Department of Anaesthesiology, All India Institute of Medical Sciences, Rajkot, Gujarat, India; 6 Department of Physiology, Teerthanker Mahaveer Medical College & Research Centre, Teerthanker Mahaveer University, Moradabad, Uttar Pradesh, India; 7 Department of General Medicine, Government Medical College, Bhavnagar, Gujarat, India; 8 Department of Surgery, Dhemaji Civil Hospital, Dhemaji, Assam, India

**Keywords:** Artificial intelligence, CRP, machine learning, postoperative infection, white blood cells

## Abstract

The application of artificial intelligence in predicting postoperative infections using routine blood parameters is of interest. Hence, a cohort of 120 surgical patients was analyzed and machine learning models were developed using WBC, CRP, NLR and other markers. The Random Forest model achieved the highest predictive performance with an AUC of 0.93. CRP and NLR were identified as the most influential predictors. Thus, we show the integration of AI for early infection detection in surgical care.

## Background:

The postoperative infection is one of the key adverse outcomes of the surgical operation, causing undesirable morbidity in patients, extended hospitalization and sometimes death. Such adverse outcomes occur early in life and early intervention is key in reducing them [1]. In traditional practice, clinicians would use a mix of a clinical manifestation, imaging and other laboratory tests to identify infections. Nevertheless, the approaches are likely to reveal infections when they have already had clinical effects and have less time to respond in time [2]. In this regard, the usefulness of Artificial Intelligence (AI) in predicting post-operative infections early using routine clinical data should be exploited [3]. Artificial intelligence (AI) technologies, specifically, machine learning (ML) algorithms, have been shown to have great potential in the medical field by discovering complex, non- linear relationships in large databases that are not clear to traditional statistical analysis [4]. Routine haematology, as with the white blood cell count, C-reactive protein (CRP), neutrophil-to-lymphocyte ratio (NLR) and other haematology markers, is traditionally gathered after surgery and offers a wealth of evidence about inflammatory and immune reactions in the body [5]. They are also pocket friendly parameters that can be easily attained and thus they can be used as inputs in AI-based predictive models [6]. Previous research studies have examined the creation of AI-based models based on these routine blood tests to predict the patients susceptible to postoperative infection across other fields of surgery such as: gastrointestinal, orthopedic and cardiothoracic surgeries [7]. Decision trees, support vector machines, random forests and neural networks demonstrated potentially accurate results to categorise the likelihood of an infection based on the early postoperative data. Also, the incorporation of temporal data (auto tracking of blood markers over time) served to increase predictive performance [8]. The fact that AI can assess risks in real time based on the data it automatically processes can assist clinicians in making informed decisions concerning the early antimicrobial treatment, further diagnostic, or extended observation [9]. Additionally, a non-invasive approach to the utilisation of the routine blood parameters is compatible with patient-centred care practice and resource-effective care delivery. Although there is potential, several obstacles still exist in translating AI models into clinical practice in terms of the generalizability of the models, data quality, interpretability and regulatory approval [10]. However, recent progress in the areas of AI interpretability and federated learning is promising to bring more transparent, robust, clinically applicable AI tools. Therefore, it is of interest to describe the effectiveness of artificial intelligence models in accurately predicting postoperative infections using routine blood parameters.

## Methodology:

The study selectively engaged 120 adult patients that underwent elective or emergency surgical operations in the general surgery, orthopedic, cardiothoracic, departments. The following inclusion criteria were used: age above 18 years, surgical intervention with a postoperative hospital stay of at least three days, provision of informed consent. Patients excluded had underlying infections, were immunocompromised (e.g., HIV-positive, receiving chemotherapy) or their laboratory data was incomplete. Clinical and demographic information were gathered, such as age, sex, comorbidities, type of surgery and duration of surgery. Routine blood parameters were taken at three stages, at the baseline (preoperative), day 1 after surgery and day 3 after surgery. These parameters were a white blood cell (WBC) count, C-reactive protein (CRP), neutrophil-to-lymphocyte ratio (NLR), platelet count, hemoglobin, total leukocyte count (TLC) and erythrocyte sedimentation rate (ESR). Postoperative infections were identified by clinical assessment, microbiological cultures (where possible) and radiology, according to the CDC criteria of diagnosing surgical site infections. The obtained data were utilized to train and test machine learning algorithms to be able to predict postoperative infections. The dataset was split up randomly into a training and test set (80% and 20%, n=96 and n=24 resp.). The method used to select the feature was recursive feature elimination and correlation matrix analysis to reduce redundancies and select the most relevant predictors. Four machine learning models were used and tested: logistic regression, random forest, support vector machine (SVM) and gradient boosting classifier. Models were generated/tested with a 5-fold cross-validation in the training set so that they are not over-stated and hence render robustness. An effort to tune hyperparameters was on the basis of grid search optimization Accuracy, sensitivity, specificity and area under receiver operating characteristic (AUC-ROC) curve the performance of this model were used. The results of the last models were analyzed on a testing set to evaluate their generalizing quality. The preprocessing of all the data and the development of the models were performed with Python and the corresponding library of machine learning (Scikit-learn and XGBoost).

## Results:

A total of 120 patients were included in the study, with a mean age of 49.6 ± 13.2 years. The sample comprised 65 males (54.2%) and 55 females (45.8%). The surgical procedures performed included gastrointestinal (GI) surgeries (40%), orthopedic surgeries (35%) and cardiothoracic surgeries (25%). These details are presented in Table 1 (see PDF). Postoperative infections occurred in 34 patients (28.3%), while the remaining 86 (71.7%) experienced no infectious complications. Comparative analysis of blood parameters between infected and non-infected groups revealed statistically significant differences. Patients who developed infections had higher levels of white blood cell count (12.8 ± 3.1 vs. 9.6 ± 2.5 x10^9^/L), CRP (88.5 ± 25.6 vs. 42.3 ± 18.9 mg/L) and neutrophil-to-lymphocyte ratio (6.9 ± 2.4 vs. 3.2 ± 1.6), all with p-values < 0.01. Platelet counts were also elevated in infected patients (320 ± 75 vs. 270 ± 68 x10^9^/L, p = 0.04). These findings underscore the association between elevated inflammatory markers and the development of postoperative infections, as shown in Table 2 (see PDF). Four machine learning models were trained and evaluated to predict postoperative infections using the collected routine blood parameters. The Random Forest classifier outperformed all other models, achieving the highest accuracy of 89.6%, sensitivity of 85.3%, specificity of 91.8% and an AUC-ROC of 0.93. Gradient Boosting followed closely, with an accuracy of 88.3% and an AUC-ROC of 0.91. Support Vector Machine (SVM) and Logistic Regression models showed lower but acceptable performance, with AUC-ROC scores of 0.81 and 0.78 respectively. The comparative performance of these models is summarized in Table 3 (see PDF). The Random Forest model's confusion matrix on the test dataset revealed 22 true positives, 2 false negatives, 2 false positives and 20 true negatives, demonstrating its strong predictive capability. Feature importance analysis confirmed that CRP on postoperative day 3, NLR and WBC count were the most influential features contributing to accurate predictions. These results demonstrate the potential of AI to effectively predict postoperative infections based on routine blood tests, enabling earlier clinical interventions and improved patient care.

## Discussion:

The current research exhibited the excellent predictive performance of the AI models, namely, the Random Forest classifier, to determine the presence of postoperative infection using a typical set of blood values, including WBC, CRP, NLR and platelet count. The results are consistent with emerging evidence that suggests the use of AI in the postoperative surveillance to present the earliest markers of infectious complications. An example is a research article by Eskandar *et al.* (2024) [11] on AI-powered risk stratification in the cases of urological malignancy. Even though not infection-specific, their results highlighted the importance of incorporating regular and clinical variables to achieve accurate risk scores with ensemble models with comparable accuracy margins of 85-90. This correlates with our findings, where Random Forest attained an accuracy of 89.6 with CRP and NLR being identified as essential variables. In a related study, Simon *et al.* (2025) [12] in Transplant International used AI to predict complications, including the risk of developing infection after undergoing lung transplantation. Their model incorporated blood gas parameters and hemodynamic data and it had a medium-to-high degree of predictive ability with AUC scores of up to 0.90. The AUC of 0.93 in our study with straight-forward, routinely available data in blood indicates that effective prediction can be achieved without requiring complex or invasive data. Transplant International proteomic extracellular vesicles improve machine-learning prediction of the outcome of liver transplant patients. In a multifaceted study on the uses of clinical AI, Alowais *et al.* (2023) [13] acknowledged the dynamically changing role of machine learning in controlling infection in the multiple specialties. Their study showed the potential of clustering and classification algorithms to take laboratory data to stratify infection risks in surgical and ICU care. We extended this to validate model architectures (Random Forest wise and Gradient Boosting) and confirm their clinical usefulness through real-world surgical data Also, most other studies possess an element of multifactorial input (imaging, genetic markers), but given the cost-effective and non-invasive nature of our markers, their usefulness is further delineated by our conceptualization. This research proved that an equivalent predictive performance may be achieved without extended variables or resource-paid diagnostics by using the standard routinely measured hematology parameters, which is particularly important when applied to resource-limited settings. Finally, most of the previous AI models have been accused of lack of interpretability, which can reduce clinical adoption. Nevertheless, we overcame this obstacle using feature importance analysis, which made it clear that the most influential predictors were CRP (primarily on postoperative day 3), NLR and WBC, contributing to transparency and trust in the conclusions between clinicians.

## Conclusion:

Routine blood parameters, artificial intelligence models and especially Random Forest are capable of predicting a postoperative infection with high accuracy is shown. The most significant predictors were the levels of RP, NLR and WBC as factors in early detection. It is possible to integrate AI in postoperative care so that timely interventions can be put in place to enhance surgical outcome.
